# Using Psychological Science to Understand and Fight Health Misinformation: An APA Consensus Statement

**DOI:** 10.1037/amp0001598

**Published:** 2025-10-20

**Authors:** Sander van der Linden, Dolores Albarracín, Lisa Fazio, Deen Freelon, Jon Roozenbeek, Briony Swire-Thompson, Jay Van Bavel

**Affiliations:** 1 Department of Psychology, University of Cambridge; 2 University of Pennsylvania; 3 Department of Psychology and Human Development, Vanderbilt University; 4 Annenberg School for Communication, University of Pennsylvania; 5 Network Science Institute, Northeastern University; 6 Department of Psychology, New York University

**Keywords:** misinformation, fake news, public health, consensus report

## Abstract

There is widespread concern that misinformation poses dangerous risks to health, well-being, and civic life. Despite a growing body of research on the topic, significant questions remain about the psychological factors that render people susceptible to misinformation, the extent to which it affects real-world behavior, how it spreads online and offline, and intervention strategies that counter and correct misinformation effectively. This report reviews the best available psychological science research to reach consensus on each of these crucial questions, particularly as they pertain to health-related misinformation. In addition, the report offers eight specific recommendations for scientists, policymakers, and health professionals who seek to recognize and respond to misinformation in healthcare and beyond.

Research on the psychology of misinformation has proliferated in recent years (e.g., Ecker et al., 2022; Lazer et al., 2018; Roozenbeek et al., 2023; Van Bavel et al., 2021; van der Linden, 2022, 2023). In 2020, the World Health Organization declared a worldwide “infodemic” (Briand et al., 2021; Tedros, 2020) based on concerns that “a global epidemic of misinformation—spreading rapidly through social media platforms and other outlets—poses a serious problem for public health” (Zarocostas, 2020, p. 1). A recent example is the spread of misinformation about the measles, mumps, and rubella vaccine, which has been associated with significant decreases in vaccine uptake (Burgess et al., 2006; Lewis & Speers, 2003; Motta & Stecula, 2021).

However, experts remain divided on many key issues, including how to best define misinformation, how to quantify how many people are regularly exposed to it, what factors make people susceptible to believing and sharing it online and offline, and how best to counter the problem at scale. These conflicting accounts can lead to confusion in the literature as well as among policymakers and practitioners, delaying or undermining appropriate action. The purpose of this report was to bring clarity to these important debates by providing a consensus view on three critical overarching questions about misinformation research, particularly as it relates to health:

1. What are the psychological factors that make people susceptible to believe and act on misinformation?
2. How and why does misinformation spread?
3. What interventions can be used to counter misinformation effectively?

To fully grasp the impact of health misinformation, it is necessary to understand the psychological factors that drive it in general: the qualities that make us likely to believe and share it, the levers of manipulation used by its creators, and the network effects induced by today’s media and political landscape. Using these insights, psychological scientists have developed and tested a broad array of methods to address and counter misinformation, many of which are examined in this report. Although not itself a systematic review, our report is based on peer-reviewed empirical studies and includes primary research articles, meta-analyses, systematic reviews, case studies, and other reports. We conclude with eight specific recommendations for scientists, policymakers, and health professionals.

## Defining Misinformation

One approach to defining misinformation has been to operationalize it at the level of the credibility of the source (e.g., Altay, Nielsen, & Fletcher, 2022; Grinberg et al., 2019). The underlying idea here is that low-credibility media outlets are likely to share more misinformation than high-credibility ones. Another approach operationalizes misinformation at the level of content, determined by whether content has been fact-checked (Pennycook & Rand, 2019) or whether claims run contrary to prevailing expert consensus (Vraga & Bode, 2020). None of these definitions are perfect, and they should be viewed as complementary rather than competing. However, the most problematic information is often not completely false but rather manipulative, biased, or otherwise misleading (Wardle, 2018).

Our definition, therefore, focuses on the extent to which a headline or claim shows evidence of manipulation, regardless of the article’s source or intent or whether it has been fact-checked. A good example is the headline: “A ‘healthy’ doctor died two weeks after getting a COVID-19 vaccine; CDC is investigating why” (Benton, 2021). This article was published by a credible outlet, the *Chicago Tribune*, and technically it is not false. However, there was no evidence at the time that the doctor died *because* of the COVID-19 vaccine, yet the headline falsely implied causation where there was only correlation (van der Linden, 2022). The article became the most shared story on Facebook in the first quarter of 2021 and is estimated to have negatively impacted vaccination attitudes to a much greater extent than fact-checked misinformation (Allen et al., 2024; van der Linden & Kyrychenko, 2024).

Another distinction is often made between “misinformation” and “disinformation,” in which the latter involves explicit intent to manipulate or deceive others (Roozenbeek & van der Linden, 2024). Motive is useful to consider, but it is often hard to prove without legal or historical documentation (Swire-Thompson & Lazer, 2020). Accordingly, we adopt the broader term “misinformation” in this report,^1^ which we define as “any information that is demonstrably false or otherwise misleading, regardless of its source or intention.”

## Susceptibility: Why Do People Believe Misinformation?

While it may be difficult to notice false information in real time, “susceptibility” to misinformation rises and falls depending on specific characteristics of the information and its audience. For example, misinformation from ingroup sources is generally more believable than misinformation from outgroup sources. One strong affinity in this regard is political alignment: Misinformation from conservative sources was rated as more accurate by conservative participants than by liberal ones, while misinformation from liberal sources was rated as more accurate by liberal participants than by conservative ones (Traberg & van der Linden, 2022). Moreover, consistent with research on persuasion indicating that beliefs about a source’s credibility influence accuracy judgments (Kumkale & Albarracín, 2004; Nadarevic et al., 2020; Pornpitakpan, 2004), credibility ratings mediated the source effects so that congenial sources were evaluated as more credible (Traberg & van der Linden, 2022).

The content of misinformation also affects belief. Americans were more likely to believe false news stories criticizing their opposing political party than those criticizing their preferred party (Pereira et al., 2021). Similarly, people in Ireland falsely remembered fake scandals more often when the scandal reflected negatively on outgroup members (Murphy et al., 2019). The emotional impact of content matters too: People were more likely to believe false statements that would make a believer happy (e.g., “Positive thoughts can cleanse the body of toxins”) compared with statements that would make one sad (e.g., “Bad things happen to certain people because they attract negative energy”; Altay et al., 2023). There is both correlational and causal evidence that inducing an emotional state can make people more susceptible to misinformation (Martel et al., 2020).

We also know that repeated information is thought to be more true, even for known falsehoods (Fazio, 2020b) and when it contradicts our prior knowledge (e.g., Fazio et al., 2015); this phenomenon is known as “illusory truth” (see Dechêne et al., 2010, for a meta-analysis). The illusory truth effects occur across age groups (Brashier et al., 2017; Fazio & Sherry, 2020) and in real-world situations such as text messages (Fazio et al., 2022; Pillai et al., 2023). Moreover, repetition drives belief in an exponential manner, with the largest increases happening during the first few exposures (Fazio et al., 2022; Hassan & Barber, 2021), suggesting that it is important to stop misinformation early.

A variety of individual differences affect susceptibility to misinformation (e.g., Nan et al., 2022). For example, higher levels of education (e.g., Albarracín et al., 2021), analytical reasoning, and numeracy skills are negatively associated with endorsement of misinformation (e.g., Bronstein et al., 2019; Pennycook & Rand, 2019; Roozenbeek et al., 2020). People who reason well with numbers and score high on measures of metacognition (e.g., actively open-minded thinking; not being overconfident in their ability to detect false headlines) tend to be better at distinguishing true versus false information (Mirhoseini et al., 2023; Saltor et al., 2023; Roozenbeek, Maertens et al., 2022; Lyons et al., 2021).

Regarding age, older adults (>65 years) are more likely than younger adults to see and share false information on social media (Grinberg et al., 2019; A. Guess et al., 2019), but, paradoxically, they are also better than younger adults at distinguishing between true and false news headlines (Brashier & Schacter, 2020; Kyrychenko et al., 2025). This effect has yet to be explained but may involve several factors associated with older adults: poor digital literacy, greater trust in news, communication goals that do not emphasize accuracy, and a larger knowledge base (Brashier & Schacter, 2020).

Researchers have noted relatively small and inconsistent correlations between the Big Five personality inventory and susceptibility to misinformation (cf. Calvillo et al., 2021; Lawson & Kakkar, 2022). Anxiety levels can predispose individuals to believe misinformation (e.g., Albarracín et al., 2021), and a 5-decade cohort study from childhood to midlife found that vaccine-hesitant individuals reported greater trauma and adverse childhood experiences fostering mistrust (Moffitt et al., 2022).

Finally, many studies have found that conservatives in the United States were more likely than liberals to believe misinformation (e.g., Baptista & Gradim, 2022; Garrett & Bond, 2021). However, it is unclear if conservatives are more psychologically vulnerable to misinformation (Jost et al., 2018; Pereira et al., 2021) or if they are more heavily targeted by misinformation (Ditto et al., 2018; A. M. Guess et al., 2020).

## Impact of Misinformation on Beliefs

Establishing the impact of misinformation requires careful attention to whether outcome measures of impact are based on beliefs, attitudes, intentions, or behaviors. The influence of misinformation on beliefs has been well established in both primary research studies and meta-analyses. These effects are typically very large across domains, including in laboratory experiments (Chan et al., 2017) and when it comes to misinformation about scientific topics (Chan & Albarracín, 2023).

## Impact of Misinformation on Attitudes

The effects of misinformation on attitudes are considerably smaller than its effects on beliefs. One laboratory experiment showed that reading about a COVID-19 conspiracy theory (vs. receiving no information at all) had a detrimental effect on institutional trust and support for government regulations (Pummerer et al., 2022). Initial stronger COVID-19 conspiracy beliefs were also linked to lower institutional trust and lower support for government regulations 2 months later (Pummerer et al., 2022) and to lower support for lockdowns 4 months later (van Prooijen et al., 2023). In short, misinformation appears to have a modest overall effect on attitudes.

## Impact of Misinformation on Behavioral Intentions

Studies suggest a link between online misinformation and health-related behavioral intentions. In early 2021, the amount of COVID-19 vaccine-related misinformation shared by Twitter users in a U.S. county predicted changes in the county’s COVID-19 vaccine hesitancy rate 2–6 days later (Pierri et al., 2022). In an experimental study, participants who read an antivaccine conspiracy theory indicated that they were less likely to immunize a fictitious child against a novel disease (Jolley & Douglas, 2014). In an randomized controlled trial, exposure to five social media posts containing misinformation about the COVID-19 vaccine led to a small decline in the number of people who would “definitely” vaccinate in both the United Kingdom and the United States (Loomba et al., 2021). Other studies, however, have shown more mixed results. For example, Pummerer et al. (2022) showed that reading a COVID-19 conspiracy theory reduced intentions toward physical distancing, but it had a much smaller effect on intentions toward safe forms of social engagement. In addition, Greene and Murphy (2021) found that exposure to false information about COVID-19 led to small reductions in intentions to vaccinate, but it had no effect on other intentions. All in all, these experiments suggest that the average impact of misinformation on intentions is small.

## Impact of Misinformation on Behavior

Some longitudinal research has assessed the effects of misinformation and conspiracy beliefs on behavior. For example, Wilson and Wiysonge (2020) looked at the impact of foreign disinformation via social media on overall vaccine uptake using global surveys and World Health Organization vaccination data from 166 countries in 2000–2018. Year over year, they found that a 1-point increase on a 5-point disinformation frequency scale was associated with a 2% point drop in the average global vaccination rate. Two meta-analyses yielded small effects of conspiracy beliefs on behavior (Bierwiaczonek et al., 2022; Stasielowicz, 2022). Interestingly, Stasielowicz (2022) found that, reciprocally, people’s pandemic-related behavior predicted their later belief in COVID-19 conspiracy theories. Using a quasi-experimental design, Carrieri et al. (2019) found that media coverage of the false autism–measles, mumps, and rubella link reduced childhood immunization rates in Italy. Finally, a systematic review of the effects of health misinformation found that misinformation negatively impacted psychological antecedents of health behavior (such as beliefs, attitudes, and intentions) in 49% of studies, but few reports directly measured real-world behaviors (Schmid et al., 2023).

## Growth: How and Why Does Misinformation Spread?

### Social and Psychological Functions of Misinformation

**Figure 1:**
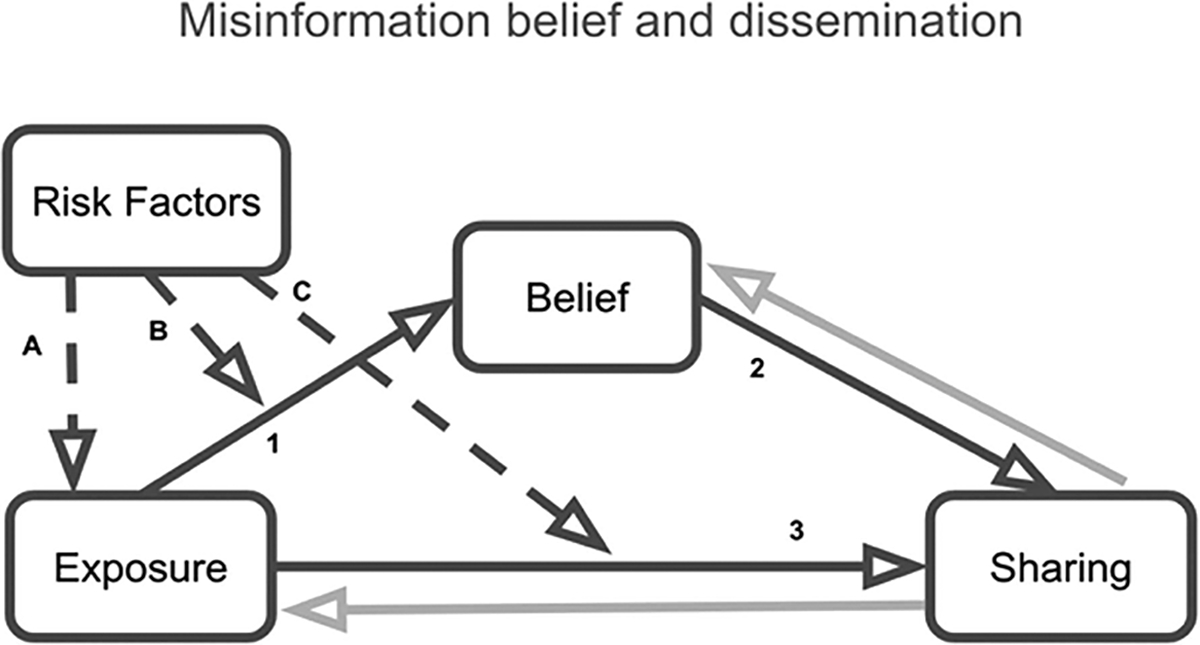
A Model of Misinformation Belief and Spread *Note.* Exposure to misinformation increases belief (Path 1) and, in turn, increases sharing (Path 2). Exposure can also increase sharing directly without affecting belief (Path 3). Psychological risk factors can increase the likelihood of exposure to misinformation (Path A); they can also affect its impact on belief (Path B) and sharing (Path C). We also propose reverse pathways for future study (gray arrows). From “Political Psychology in the Digital (Mis)Information Age: A Model of News Belief and Sharing,” by J. J. Van Bavel, E. A. Harris, P. Pärnamets, S. Rathje, K. C. Doell, and J. A. Tucker, 2021, *Social Issues and Policy Review, 15*(1), p. 86 (https://doi.org/10.1111/sipr.12077). Reprinted with permission.

Effective responses to misinformation require a detailed understanding of the social and psychological factors that drive people to spread it (e.g., Van Bavel et al., 2021; van der Linden, 2023). Figure 1 shows a model of the relationship between psychological risk factors and the spread of misinformation (Van Bavel et al., 2021). The model proposes that exposure to misinformation increases belief (Path 1), which in turn increases sharing (Path 2). This path may explain why some groups in society who are exposed to high levels of misinformation become more involved in its spread (González-Bailón et al., 2023; A. Guess et al., 2019). At the same time, people may share misinformation independently of whether they believe it (Path 3; Pennycook & Rand, 2021). For instance, people willingly spread misinformation they know is false when they expect to receive social rewards (Ren et al., 2023) or because they think it is interesting (Altay, de Araujo, & Mercier, 2022) or entertaining (van Prooijen et al., 2022). The model also describes how psychological risk factors can increase exposure to misinformation (Path A) and modulate its impact on belief (Path B) and sharing (Path C).

### Psychological Factors Driving Engagement With Misinformation

#### Partisanship

Psychologists have observed that people maintain certain beliefs long after contrary evidence proves them false (Path B, e.g., Ross et al., 1975). Although backfire effects, in which people “double down” on their initial beliefs when they are refuted, are fairly rare (e.g., Swire-Thompson et al., 2020, 2022; Wood & Porter, 2019), when information aligns with a cherished identity or worldview, people tend to interpret it in a biased manner that reinforces original predispositions. This effect is called partisan bias (Meffert et al., 2006). If the value people place on their identity is higher than the value they place on accuracy, it can lead them to believe and spread misinformation (Rathje et al., 2022; Van Bavel & Pereira, 2018). Partisan bias can arise from selective news exposure but also from prior beliefs of the individual (i.e., motivated cognition, e.g., Festinger et al., 1956; Kunda, 1990).

Political views can lead partisans to either accept misinformation or dismiss accurate news as false (Path B; Schulz et al., 2020). A recent analysis found that partisan-motivated cognition (Van Bavel & Pereira, 2018) was the single best model to account for misinformation sharing (e.g., Borukhson et al., 2022). Misinformation flourishes during periods of heightened polarization, including the run-ups to elections (Silverman, 2016), so polarization elevates risk across all stages of our model.

Even when information is implausible or clearly false, extreme partisans may choose to spread it to support their ingroup or destabilize their opponents (Path C). Recent studies indicated that people who share information in polarized environments care less about its accuracy and more about its alignment with their partisan beliefs (Osmundsen et al., 2021; see also Rathje et al., 2021). Moreover, in comparison with positive feelings toward their party, a person’s negative feelings toward their outgroup party appear to be more likely to drive sharing behavior (Osmundsen et al., 2021).

In many cases, people avoid sharing misinformation because they feel that doing so could harm their reputation (Altay et al., 2020). However, individuals with strong political views update their beliefs based on cues from both political leaders and peers (Hahnel et al., 2020; see also Zawadzki et al., 2020), and social norms operating within communities appear to moderate belief and trafficking in misinformation (Pretus et al., 2023).

#### Emotion

Another contributing factor in the belief and spread of misinformation is emotion. A recent systematic review of the literature on health misinformation found that “misinformation contained more emotion-based arguments and rhetoric compared to factual information” in 14 of the 15 included studies (Peng et al., 2023, p. 2137). One study analyzed the spread of over 125,000 true and false news stories shared on Twitter by ~3 million people from 2006 to 2017; its main finding was that misinformation diffused deeper, faster, and farther than fact-checked true information. Importantly, misinformation elicited greater surprise, fear, and disgust than did true information (Vosoughi et al., 2018), consistent with experiments in which induced emotional states were associated with increased belief in false news (Path B; Martel et al., 2020). Misinformation is known to exploit outrage online at the cost of accuracy (McLoughlin et al., 2024). That said, recent work indicates that social media diffusion patterns vary with the specific platform used (cf. Cinelli et al., 2021).

### Misinformation Spread on Legacy and Social Media

Both legacy media (TV, radio, newspapers) and social media are powerful vectors for the transmission of misinformation. However, misinformation spreads differently within each system.

#### Legacy Media

In democratic countries, mainstream news outlets generally attempt to adhere to traditional journalistic values such as accuracy, viewpoint neutrality, timeliness, and editorial independence. However, one way in which news outlets spread misinformation is through errors that squeeze past these safeguards. For instance, an Associated Press story about the arrival of Chinese-produced COVID-19 vaccines in Hungary stated: “This story has been corrected to show that about 500,000 people have been vaccinated in Serbia, including ethnic Hungarians, not 500,000 ethnic Hungarians” (Spike, 2021). The suggestion that certain ethnic groups are preferred for vaccination or withheld from it could fuel vaccine-related conspiracy theories (e.g., Albarracín et al., 2021).

Journalists can (unintentionally) abet the agendas of those who deliberately spread misinformation, for instance, in celebrity-focused “soft” news (Bruns et al., 2022). These incidents may sometimes originate in media manipulation campaigns by bad-faith actors (Benkler et al., 2018; Marwick & Lewis, 2017). Health and medical reporters generally avoid being misled by relying on medical professionals with proven track records of scientific expertise, but sometimes this approach is unsuccessful, as with widespread coverage of the spurious link between the measles, mumps, and rubella vaccine and autism (Burgess et al., 2006; Clarke, 2008; Lewis & Speers, 2003). False claims spread or repeated by trustworthy or mainstream outlets are likely to cause more damage than those promoted by fringe sources (Traberg, 2022; Tsfati et al., 2020).

#### Social Media

Unlike legacy media, social media lacks prepublication oversight as an industry standard to ensure information quality (although some platforms have safeguards in place, e.g., Kreiss, 2016). Thus, social media appeals to producers of misinformation, and some popular misinformation creators even hail the power of social media to monetize their efforts.

Social media platforms facilitate the spread of misinformation through peer-to-peer content sharing. Their low-friction network structures allow ordinary users to distribute (mis)information to much larger audiences than their creators can on their own. This idea is especially important because one of the major paths to viral visibility is through trusted influencers like celebrities and prominent politicians (Brennen et al., 2020; I. Shin et al., 2022). When influencers share messages containing misinformation, they also convey the impression that they endorse the misinformation or at least believe it is worthy of consideration (Metaxas et al., 2015).

The third major way that social media enables the spread of misinformation is via echo chambers and algorithmic filtering. Echo chambers occur when there is both homophily (i.e., “birds of a feather flock together”) and polarization in a network, which makes communities with similar beliefs or interests cluster together. Numerous studies have shown that echo chambers exist within specific social media platforms (e.g., Cinelli et al., 2020; Del Vicario et al., 2016), though scholarly debate continues over their prevalence and boundaries (e.g., Eady et al., 2019). A recent systematic review of the echo chamber hypothesis (Terren & Borge-Bravo, 2021) suggests that the ability to identify echo chambers depends on the method used: Only five of 55 studies found no evidence of echo chambers, and all five studies were based on self-reported rather than digital trace data. However, most of the digital trace studies sampled (44 of 55) relied on data from only one social media platform, and reviews of multiplatform studies continue to raise important questions about their prevalence (Bruns, 2019; Cinelli et al., 2021; A. Guess et al., 2018).

Despite these diverging conclusions, evidence indicates that the presence of social media echo chambers can facilitate the spread of misinformation (Del Vicario et al., 2016; Törnberg, 2018) and impede the spread of corrections (Zollo et al., 2017). Algorithmic filtering may also play a role: Most social media platforms use filters based on engagement data (including numbers of clicks, shares, and comments) and users’ individual platform interaction histories to determine or prioritize what content to show to users (Maréchal & Biddle, 2020). Content that exhibits negative emotions—including most misinformation (Brady et al., 2020; McLoughlin et al., 2024; Rathje et al., 2021; Solovev & Pröllochs, 2022)—tends to be promoted and recommended to users by social media platforms (Hussein et al., 2020; J. Shin & Valente, 2020; Yesilada & Lewandowsky, 2022). However, many studies lack access to user recommendations due to methodological difficulties (but see Chen et al., 2023), so our ability to fully understand the issue remains limited.

### Response: Interventions to Counter Health Misinformation

Researchers have increasingly explored how to manage and prevent exposure to misinformation and the subsequent sharing of it. Roozenbeek et al. (2023) identified two dimensions of misinformation interventions: System-level approaches that focus on achieving systemic changes (e.g., legislation, transparency standards; see also Roozenbeek & Zollo, 2022), and individual-level approaches that focus on changing individual behavior. It is possible that system-level interventions could be more effective than individual-level ones in curbing the spread of misinformation—for example, by reducing the harmful effects of recommender algorithms, demoting misinformation in online search platforms, or removing content in predatory journals from medical databases (Swire-Thompson & Lazer, 2022, but see A. M. Guess et al., 2023). However, individual-level interventions have fewer potential ramifications for freedom of expression and rely less on the ability and willingness of technology companies to combat harmful content (see Kozyreva et al., 2024; Roozenbeek et al., 2023). In this section, we therefore focus on four types of individual-level interventions: debunking, prebunking, digital literacy, and nudges.

### Debunking

Debunking or fact-checking is the correction of misinformation (Lewandowsky et al., 2020); it also involves addressing why the misinformation is incorrect and/or providing accurate information (Ecker et al., 2022). This intervention is deployed after people have been exposed to misinformation and believe it or are unsure of its veracity.

#### Efficacy of Debunking

Meta-analyses generally show that debunking is effective at reducing, but not eliminating, misperceptions (Chan & Albarracín, 2023; Chan et al., 2017). Findings are mixed as to whether health misinformation is easier to correct than political misinformation (Chan & Albarracín, 2023; Vraga et al., 2019), but Walter and Murphy (2018) posited that health misinformation may be easier to correct because topics that involve political identity are especially resistant to belief change. Debunking is most effective when a detailed reason is offered to explain why the misinformation is incorrect (Chan & Albarracín, 2023; Ecker et al., 2010; van der Meer & Jin, 2020). Debunking appears to be effective in real-world settings and across cultures. For instance, Porter and Wood (2021) found fact-checks to be effective in Argentina, Nigeria, South Africa, and the United Kingdom.

It also seems that debunking is robust to variations in how the correction is presented. Evidence suggests that corrections were equally effective regardless of their tone (i.e., uncivil, affirmational, or neutral; Bode & Vraga, 2021), whether the correction appeared to be from an algorithm or another user (Bode & Vraga, 2018), where the corrections were presented (i.e., the “related articles section” of a social media platform; Smith & Seitz, 2019), or their order (i.e., misinformation first vs. fact first; Swire-Thompson et al., 2021). It appears that simply getting people to interact with corrections is the most important component of a successful debunking strategy (Vraga & Bode, 2018).

Few studies on fact-checking include long-term measures of efficacy (Dias & Sippitt, 2020). Kowalski and Taylor (2017) showed that debunking remained partially effective and did not return to baseline for up to 2 years. However, it is well documented that the new knowledge acquired with debunking fades over time, a phenomenon known as “belief regression” (Carey et al., 2022; Swire-Thompson et al., 2023). The primary reason that belief regression occurs is that people forget the correction (Swire-Thompson et al., 2022) or they forget that the source is credible (Albarracín et al., 2017). Thus, repeated fact-checks may be particularly effective.

#### Limitations of Debunking

A primary limitation of debunking is that corrections typically reduce belief in misinformation, but not to the same extent as for people who never encountered the misinformation in the first place. Known as the “continued influence effect” of misinformation (Chan et al., 2017; Lewandowsky et al., 2012; Walter & Tukachinsky, 2020), this robust phenomenon occurs either because people fail to fully integrate the correct information into their mental model or because they fail to retrieve the correct information (Ecker et al., 2022; Sanderson & Ecker, 2020).

A second limitation is that fact-checks often fail to reach their intended targets (Zollo et al., 2017), in part because individuals who are predisposed to believe in the original misinformation actively avoid its correction (Hameleers & van der Meer, 2019).

Finally, fact-checking is a time-consuming process in which each misconception is examined individually, so there is an asymmetry between how quickly misinformation can be produced and spread and how quickly people can fact-check it. Allgaier and Svalastog (2015) also highlighted that debunking may not be a one-size-fits-all approach and may not be equally effective for every population. They suggest that if fact-checks are developed with broader sociocultural contexts in mind, they may be more effective.

### Prebunking and Psychological Inoculation

Prebunking is an umbrella term for a category of interventions intended to prevent people from believing misinformation in the first place. The method most commonly used to prebunk misinformation is psychological inoculation. According to inoculation theory (Compton et al., 2021; W. McGuire, 1964; W. J. McGuire & Papageorgis, 1961; van der Linden, 2023), exposure to a weak version of a (false) claim builds psychological resistance against future undue influence and persuasion. Psychological inoculations have two parts: a forewarning about an impending attack on a belief (e.g., “warning: people may try to manipulate you by saying X”) and a statement that preemptively refutes the falsehood (e.g., “this is not true, because Y”). In the context of misinformation, there are two dominant types of inoculation interventions: *Issue-based* interventions tackle individual claims or stories that are false, and *technique-based* interventions address the common tropes and techniques that underlie many types of misinformation (e.g., logical fallacies, emotional manipulation, conspiratorial reasoning; Compton et al., 2021; Traberg et al., 2022).

Within inoculation research, there is one additional relevant distinction, that is, between passive and active inoculation (W. McGuire, 1964; Traberg et al., 2022). Passive inoculation interventions offer participants a preemptive counterargument to misinformation, while active inoculation interventions ask participants to generate their own counterarguments. Passive inoculation interventions can be text based (Basol et al., 2021; Cook et al., 2017) or video based (Lewandowsky & Yesilada, 2021; Piltch-Loeb et al., 2022; Roozenbeek, van der Linden, et al., 2022). Active inoculation interventions often come in the form of a game or quiz (Cook et al., 2023; Roozenbeek & van der Linden, 2019).

#### Efficacy of Prebunking

Inoculation interventions have been shown to be effective at reducing susceptibility to both individual examples of misinformation (e.g., van der Linden et al., 2017) and various manipulation techniques (Traberg et al., 2022). Successful prebunking has occurred with text-based (Cook et al., 2017; Green et al., 2022), video-based (Piltch-Loeb et al., 2022), and game-based interventions (Basol et al., 2021; Cook et al., 2023; Roozenbeek, Traberg, & van der Linden, 2022). A recent systematic review and meta-analysis found that inoculation interventions are effective in creating more resistant attitudes against misinformation while improving truth discernment (Lu et al., 2023). Inoculation interventions can also protect vaccination intentions (Piltch-Loeb et al., 2022). There are few reports that directly compare passive versus active inoculation interventions (cf. Basol et al., 2021; Green et al., 2022; Maertens et al., 2025). A recent systematic review revealed that prebunking interventions had larger effect sizes than debunking interventions for countering conspiracy theories, noting that “prevention is the best cure” (O’Mahony et al., 2023, p. 14).

Maertens et al. (2021, 2025) and Basol et al. (2021) looked at the long-term effects of inoculations and found that intervention effects that dampen the perceived reliability of misinformation remained significant for at least 1 week and in some cases longer; they lasted up to 3 months or more when people were given brief reminders of the inoculation (so-called booster shots).

#### Limitations of Prebunking

Prebunking interventions are “boosts” (Hertwig & Grüne-Yanoff, 2017) in that they seek to improve the public’s ability to identify misinformation. Thus, people have to opt into taking part in the intervention. Cross-cultural adaptation and testing are lacking (Ali & Qazi, 2023), especially outside of North America and Western Europe (but see Badrinathan, 2021; Harjani et al., 2023; Iyengar et al., 2022). Real-world prebunking campaigns are also lacking. In a field study on YouTube that has since been scaled by Google across millions of social media users (Jigsaw, 2023), Roozenbeek, van der Linden, et al. (2022) showed that video-based inoculation interventions improved recognition of key manipulation techniques—but few other field studies are available, and none test behavioral measures such as sharing misinformation. However, one study did find evidence that inoculation reduced behavioral engagement with misinformation (e.g., liking, sharing) in a simulated social media setting (McPhedran et al., 2023).

In addition to their effect on misinformation, some prebunking interventions may slightly reduce the perceived reliability of more ambiguous “real news” items (Modirrousta-Galian & Higham, 2023), though a recent meta-analysis concluded that inoculation interventions do improve truth discernment overall (Lu et al., 2023). Nonetheless, any intervention may engender a degree of general skepticism about news media (Clayton et al., 2020; A. M. Guess et al., 2020; Hoes et al., 2024). However, it is possible that general skepticism is a methodological artifact of how efficacy studies are designed. For example, increased skepticism can occur when the study contains more false than true stimuli (Altay et al., 2025). There is debate about whether generalized skepticism (but not cynicism) is a good or a bad trait. Reputable sources sometimes use manipulation, clickbait, or sensationalism when presenting news, so if people become slightly less certain that a (mostly) true headline is accurate if it is presented in a biased manner, the overall result may be healthy (rather than immutable) skepticism. Finally, research has found that undesirable skepticism can be counteracted by giving people feedback on their performance, which helps promote better discernment (Leder et al., 2023).

### Health, Media, and Digital Literacy

The distinction between health literacy, media literacy, and digital literacy is increasingly blurred. Health literacy can generally be considered as the competencies required to find and evaluate health content for quality or accuracy (Norman & Skinner, 2006), media literacy focuses on the ability to evaluate print and online media messages (Potter, 2020), and digital literacy is defined as the skills required to execute tasks online (Reddy et al., 2022). Literacy interventions are often provided as part of formal education or courses in the wider community (Nygren & Guath, 2022).

#### Efficacy of Literacy Interventions

Several meta-analyses have investigated health literacy interventions. For example, Nordheim et al. (2016) conducted a systematic review of school-based interventions to enhance adolescents’ abilities to critically appraise health claims: They generally found short-term benefits on knowledge and relevant skills. Moving outside the classroom, Nutbeam et al. (2018) reviewed studies on community interventions to improve health literacy and found only seven studies that met their inclusion criteria (out of an initial pool of 1,117 articles). They concluded that the current interest surrounding health literacy was not matched by the number of systematic studies being conducted and the evidence supporting the implementation of national policies and programs was not emerging as quickly as needed. It is also possible that studies yielding nonsignificant findings remain unpublished (i.e., the “file drawer problem”).

Several meta-analyses have found media literacy interventions to be effective for improving media literacy skills (Vahedi et al., 2018), media knowledge, and critical perceptions toward media messaging or advertising (Jeong et al., 2012). However, when focusing specifically on the discernment of health misinformation, findings appear to be mixed. For example, Badrinathan (2021) found that their 1-hr media literacy intervention in India did not lead to improvements in the ability to discern health misinformation, and Vraga et al. (2021) found no effect of a news literacy video on protecting people against health misinformation. However, it is possible that the length of these interventions was too short. Bergsma and Carney (2008) found in a meta-analysis of 28 health-promoting media literacy interventions that long interventions (5 hr or more) were more likely to be effective than those that were short (<60 min). In addition, some interventions may work best in conjunction with others. For instance, Hameleers (2022) found that media literacy paired with fact-checking was more effective than either intervention alone in samples from both the United States and the Netherlands.

Although digital literacy interventions have been studied much less than health-focused or media-focused efforts, promising research is emerging. For example, A. M. Guess et al. (2020) found that digital literacy training helped individuals distinguish between mainstream and false news in both the United States and among highly educated Indian participants. Moore and Hancock (2022) found that digital literacy training improved fake news discernment in older adults. Digital literacy can also improve online reasoning (McGrew et al., 2019; Nygren & Guath, 2022) and lateral reading on social media (Panizza et al., 2022).

Reviews and meta-analyses of health and media literacy interventions have long highlighted the lack of research on their efficacy over time. Bergsma and Carney (2008), Manafo and Wong (2012), and Nordheim et al. (2016) found in their reviews that there were beneficial short-term effects of health-promoting media literacy interventions, but no studies evaluated the long-term effects of such interventions. However, studies have recently begun to investigate these interventions with delayed retention intervals. Stassen et al. (2020) conducted a pre-/post-randomized controlled trial via a web-based health literacy intervention and included a 6-month follow-up, but they found that their 8-week intervention did not increase health literacy when compared with a control group, either immediately or at follow-up. Digital literacy interventions by A. M. Guess et al. (2020) and McGrew et al. (2019) found effects that persisted after a 3-week period, but these improvements faded over time.

#### Limitations of Literacy Interventions

One limitation for health, media, and digital literacy interventions is that they are often quite lengthy and commonly require cooperation from schools, school districts, community centers, and/or local and national governments. Another potential limitation is cross-cultural applicability: Badrinathan (2021) and A. M. Guess et al. (2020) tested interventions on rural samples in India and found that their interventions were broadly ineffective. The largest problem with evaluating the efficacy of these interventions is that they vary widely in terms of content and duration, from a couple minutes to multiple weeks (Stassen et al., 2020). Finally, studies are hard to compare because of different outcome measures (Smith et al., 2021), so the field should consider establishing consensus on appropriate outcomes rather than using customized measures that vary from study to study (Nutbeam et al., 2018).

### Nudging

Thaler and Sunstein (2008) defined nudges as “any aspect of the choice environment that alters people’s behavior in a predictable way without forbidding any options or significantly changing their economic incentive” (p. 6). Nudging interventions against misinformation are designed to positively influence people’s behavior by, for example, prompting them to share less misinformation or low-quality content on social media. Major advantages of these interventions include that they are relatively easy to implement on social media and they do not require people to opt into the intervention.

Several antimisinformation nudges have been proposed. *Accuracy prompts* involve making the concept of accuracy more salient in people’s minds, which should then improve the quality of the content they share with others (Pennycook et al., 2021). *Social-norm nudges* are geared toward news-sharing behavior and emphasize either injunctive norms (i.e., behaviors most people find acceptable or not) or descriptive norms (i.e., how other people respond in certain situations). *Motivational nudges* seek to motivate people to be as accurate as possible (e.g., paying them to correctly identify true and fake news; Rathje et al., 2023). Other types of nudging interventions exist as well, such as asking people to pause to consider the accuracy of headlines (Fazio, 2020a).

#### Efficacy of Nudging

Pennycook et al. (2020) found that a single accuracy prompt improved “sharing discernment,” a measure of the quality of people’s news-sharing decisions, for true versus false news headlines about COVID-19. An internal meta-analysis by the same team (Pennycook & Rand, 2022) found that accuracy nudges were effective overall at improving sharing discernment, although this effect was small and did not occur in all of the studies included. The effect appeared to be stronger for more intensive interventions (e.g., multiple prompts shortly after one another) and weaker for a one-off accuracy prompt (Pennycook & Rand, 2022). A cross-cultural study in 16 countries showed that accuracy improved the quality of people’s sharing intentions in some countries, but not in countries where people professed higher belief in misinformation (Arechar et al., 2023). Nonetheless, a field study on Twitter showed that a nudge to share information from higher quality news sources (e.g., *The New York Times*, CNN) led to improvements in the quality of the sources people shared (Pennycook et al., 2021). Motivational nudges (e.g., paying people to be as accurate as possible) significantly boosted discernment and reduced partisan bias in people’s assessments of news headlines, mainly because people who were motivated to be accurate were more likely to identify true news stories that were incongruent with their political beliefs as correct (Rathje et al., 2023).

There is some ambiguity when it comes to the longevity of the nudging effect. In their field study, Pennycook et al. (2021) found that a single accuracy prompt was effective over a 24-hr period in improving the quality of news content shared. Roozenbeek et al. (2021), on the other hand, found some evidence for rapid decay, as the nudging effect in their study appeared to have worn off after several headline evaluations.

#### Limitations of Nudging

Nudges appear to become less effective the more often people are exposed to them (Sasaki et al., 2021), but it is unclear if this is the case for all types of nudges. Some people do not respond to nudges, especially when they do not want to be nudged, a concept known as “nudgeability” (de Ridder et al., 2021). In addition, the replicability of accuracy nudge interventions appears to be somewhat mixed: Roozenbeek et al. (2021) initially failed to replicate the aforementioned COVID-19 accuracy nudge study by Pennycook et al. (2020), but they found a small effect after collecting additional data. Accuracy nudges had no effect on a sample of U.S. conservatives and Spanish far-right voters (Pretus et al., 2023), and several other articles have reported failed or mixed replications (e.g., Gavin et al., 2022). One explanation for this inconsistency is that nudges may work less well for persuasive misinformation or for people who often rate misinformation as accurate (Arechar et al., 2023; Pennycook & Rand, 2022; Roozenbeek et al., 2023).

### Recommendations

#### Recommendation 1: Avoid Repeating Misinformation Without Including a Correction

The repetition of false claims increases belief in those claims. This phenomenon, known as the illusory truth effect, affects people of all ages, even when they already have relevant prior knowledge about the topic. Repeating misinformation is necessary only when actively correcting a falsehood. In these cases, the falsehood should be repeated briefly, with the correction featured more prominently than the falsehood itself.

### Recommendation 2: Collaborate With Social Media Companies to Understand and Reduce the Spread of Harmful Misinformation

Most misinformation on social media is shared by very few users, even during public health emergencies. These “superspreaders” can play an outsized role in distributing misinformation. Social media “echo chambers” bind and isolate communities with similar beliefs, which aids the spread of falsehoods and impedes the spread of factual corrections. On social media, sensational, moral–emotional, and derogatory content about the “other side” can spread faster than neutral or positive content. Scientists, policymakers, and public health professionals should work with online platforms to understand and harness the incentive structures of social media to reduce the spread of dangerous misinformation.

### Recommendation 3: Use Misinformation Correction Strategies With Tools Already Proven to Promote Healthy Behaviors

There is strong evidence that curbing misperceptions can change underlying health-related beliefs and attitudes, but it may not be sufficient to change real-world behavior and decision making. Correcting misinformation with accurate health guidance is vital, but it must happen in concert with evidence-based strategies that promote healthy behaviors (e.g., counseling, skills training, incentives, social norms).

### Recommendation 4: Leverage Trusted Sources to Counter Misinformation and Provide Accurate Health Information

People believe and spread misinformation for many reasons: They may find it consistent with their social or political identity, they may fail to consider its accuracy, or they may find it entertaining or rewarding. These motivations are complex and often interrelated. Attempts to correct misinformation and reduce its spread are most successful when the information comes from trusted sources and representatives, including religious, political, and community leaders.

### Recommendation 5: Debunk Misinformation Often and Repeatedly Using Evidence-Based Methods

Debunking misinformation is generally effective across ages and cultures. However, debunking typically does not eliminate misperceptions completely. Corrections should feature prominently with the misinformation so that accurate information is properly processed and later retrieved. Debunking is most effective when it comes from trusted sources, provides sufficient detail about why the claim is false, and offers guidance on what is true instead. Because the effectiveness of debunking fades over time, it should be repeated through trusted channels and evidence-based methods.

### Recommendation 6: Prebunk Misinformation to Inoculate Susceptible Audiences by Building Skills and Resilience From an Early Age

Instead of correcting misinformation after the fact, prebunking should be the first line of defense to build public resilience to misinformation in advance. Psychological inoculation interventions can help people identify individual examples of misinformation or the overarching techniques commonly used in misinformation campaigns. Prebunking can be scaled to reach millions on social media with short videos or messages, or it can be administered in the form of interactive tools involving games or quizzes. However, the effects of prebunking fade over time; regular “boosters” may be necessary to maintain resilience to misinformation, along with media and digital literacy training.

### Recommendation 7: Demand Data Access and Transparency From Social Media Companies for Scientific Research on Misinformation

Efforts to quantify and understand misinformation on social media are hampered by the lack of access to user data from social media companies. Misinformation interventions are rarely tested in real-world settings due to a similar lack of industry cooperation. Publicly available data offer a limited snapshot of exposure, but they cannot explain population and network effects. Researchers need access to the full inventory of social media posts across platforms, along with data revealing how algorithms shape what individual users see. Responsible data sharing could use frameworks currently in use to manage sensitive medical data. Policymakers and health authorities should encourage research partnerships and demand greater oversight and transparency from social media companies to curb the spread of misinformation.

### Recommendation 8: Fund Basic and Translational Research Into the Psychology of Health Misinformation, Including Effective Ways to Counter It

Several interventions have been developed to counter health misinformation, but researchers have yet to compare their outcomes, either alone or in combination. There is a need to understand which interventions are effective for specific types of information: What works for vaccine misinformation may not translate to misinformation about cancer. Ideally, these questions would be answered by large-scale trials with representative target audiences in real-world settings. Increased funding opportunities for psychological science research are needed to address these important questions about digital life.

## Public Significance Statement

The spread of misinformation poses risks to individual health and societal well-being. However, many questions remain about how and why misinformation spreads, how it impacts behavior, and how best to counter it. This consensus report outlines the best available psychological science on (health) misinformation. It offers eight concrete recommendations to help scientists, policymakers, and health professionals respond to the ongoing threats posed by misinformation.

## Footnotes

1 The exception is when we refer to documented disinformation campaigns wherein intent has clearly been established.
